# The local cellular response to Human Papillomavirus focuses on basal layer restoration as visualized *in situ* by specific cellular neighborhoods near infected cells

**DOI:** 10.3389/fimmu.2025.1728629

**Published:** 2025-12-08

**Authors:** Roderick C. Slieker, Ziena Abdulrahman, Saskia Santegoets, Mariette I. E. van Poelgeest, Marij J. P. Welters, Sjoerd H. van der Burg

**Affiliations:** 1 Department of Medical Oncology, Oncode Institute, Leiden University Medical Center, Leiden, ZH, Netherlands; 2 Department of Gynecology, Leiden University Medical Center, Leiden, Zuid-Holland, Netherlands

**Keywords:** CosMx, spatial transcriptomics, VIN, vHSIL, neighborhoods

## Abstract

**Background:**

Human papillomavirus type 16 (HPV16) is known to deregulate and cause hyperproliferation of the infected epithelium, but its full effect on the host’s tissue has remained elusive as earlier comprehensive studies were restricted to *in vitro* models.

**Aim:**

The aim of the current study was to study HPV16-induced tissue changes in vulvar tissue in its full natural context by using single-cell spatial transcriptomics.

**Methods:**

We applied spatial single-cell spatial transcriptomics to formalin-fixed paraffin-embedded healthy vulvar tissue (n=5) and HPV16+ high grade vulvar lesions (vHSIL, n=31).

**Results:**

More than 415,000 individual cells were identified and annotated *in situ*, to create complete digital atlases of the healthy vulvar and HPV16+ vHSIL tissues for comparison. Two subpopulations of basal cells were identified, one of which lined the basal membrane of healthy tissue, the other is characterized by a transcriptomic signature like HPV16-infected keratinocytes and upregulated inflammatory genes and observed predominantly in vHSIL. Epithelial cells in the parabasal layers of vHSIL upregulated the expression of genes associated with inflammation and proliferation. Importantly, HPV16 profoundly remodeled stromal fibroblasts, endothelial, and immune cells, reorganizing them into distinct cellular neighborhoods of which three dominated vHSIL. A regenerative niche adjacent to the basal layer, a perilesional niche characterized by immune suppression, and a more distal niche enriched for adaptive immune activity.

**Conclusion:**

In conclusion, spatial analysis reveals that HPV16 infection orchestrates a coordinated tissue response, driving basal layer restoration while locally suppressing immune activation to prevent pathological damage.

## Introduction

Human Papillomavirus type 16 (HPV16) infects the basal cell membrane of the vulvar epithelium (1). The DNA of HPV encodes multiple oncogenes that collectively contribute to the deregulation of the basal epithelial cells and hyperproliferation of epithelial cells via dysregulation of cell cycle regulators including Rb and p53 (1). Ultimately, HPV16-mediated changes lead to the development of cancer.

Previous studies have investigated the effects of the HPV16 encoded early proteins – mostly limited to E6 and E7 oncogenes – in keratinocytes but were limited to *in vitro* models based on micro-arrays or next generation sequencing (2–4) or in organoids combined with single cell RNA-sequencing to model the changes that occur upon HPV16 infection (5). However, high dimensional single-cell spatial transcriptomics now provides the possibility to study HPV16-induced changes in its full natural context. This allows the detection and visualization of changes not only in the epithelial cells but on all different types of cells present in the tissue microenvironment at the single cell resolution, using formalin-fixed paraffin-embedded (FFPE) archived material. Most recently, this technique was used to determine differences in the presence and organization of immune cell infiltration in the context of immunotherapy of vulvar high grade squamous intraepithelial lesions (vHSIL (6)).

Here, we applied single-cell spatial transcriptomics to generate a cell atlas of the vulvar microenvironment and to investigate and visualize the impact of HPV16-infection on the vulvar tissue by comparing HPV16+vHSIL with healthy vulvar tissue. This led to an unprecedented insight into biological context governing the disease process of an epithelial infection with HPV16. High numbers of HPV16-infected basal cells visibly disturbed the basal membrane with, in return, a host’s response of different cell types, focused on restoration of the basal layer and very local suppression of the adaptive immune response that is aroused to prevent pathological damage.

## Methods

### Human tissue samples

Formalin-fixed paraffin-embedded (FFPE) tissue sample blocks of healthy HPV-negative vulva (n=5) and HPV16+ vulvar high-grade squamous intraepithelial lesions (vHSIL, n=20, **Supplementary Table S1**) were analyzed by CosMx 1000-plex spatial transcriptomics. The presence of HPV was assessed either by using 3 sets of general primers for HPV (CPI/II, MY 9/11, GP 5+/6+) which was then followed by sequencing, or by an HPV16-specific PCR followed by electrophoreses of the product to confirm the presence of one product. In addition, from n=11 patients a biopsy was available upon therapeutic HPV16-SLP vaccination (7, 8), this was not available for all patients, as some had already cleared the vHSIL lesion by then. The study was conducted in accordance with the Declaration of Helsinki. The study was approved by the national Central Committee on Research Involving Human Subjects (CCMO, NL21215.000.08) and the Leiden University Medical Center institutional ethical committee (P08.197). Informed consent was given by the participants before taking part.

### Spatial transcriptomics and cell annotation

CosMx spatial transcriptomics data was generated for five healthy vulva tissues and 31 HPV+ vHSIL. Part of data of HPV16+ lesions was generated before (6). Slides were stained for PanCK, DAPI, CD68 and CD45 using immunofluorescence. Cell segmentation was performed using Cellpose within the Nanostring AtoMx environment. Cell annotation was performed using an in-house R package *CosMax* and figures produced with an in-house dashboard *CosMXplorer*. Cell annotation was performed by using reference profiles and external datasets implemented in the *CosMxData* R package to predict B-cells, endothelial cells, fibroblasts, macrophages, mast cells, mDCs, pDCs, monocytes, neutrophils, NK cells, plasma cells, T CD4+ and T CD8+ cells. When insufficient cells were found for accurate prediction, cell types were excluded. Provided that additional cell types will be present in addition to the aforementioned cell types, we allowed 15 additional clusters to be identified. We compared annotation of cells to annotation on the basis of three external datasets from Kong et al, Conde et al, and Ji et al. (9, 10)).

### Cell subclustering

To adjust for the batch effects between the datasets, we used SCT normalization to regress out the batch effects due to slide-to-slide variation. Given that cell subclustering will be influenced by transcripts bleeding from neighboring cells, we used a contamination metric as implemented in the function “*pre_de”* from the *smiDE* package (11). Genes whose expression was most likely the result of bleeding from nearby cells were excluded for that cell type, then dimension reduction and Louvain clustering was performed on the smaller set comprised of the single cell type. Cell subclusters were further annotated by comparing them to previously published atlases of cell types including macrophages, endothelial cells (12), fibroblasts (13, 14), plasma cells (15), T CD4+ cells (16), mast cells (17), B-cells (18), and neutrophils (19).

### Differentially expressed genes and enrichment

Differentially expressed genes were identified using the *FindMarkers* or *FindAllMarkers* function from the Seurat package. Genes were considered differentially expressed when the adjusted P-value was below 0.05, the log fold change (logFC) above or below 0, and the percentage of cells expressing the gene in the group of interest above 25%. Enrichment was performed based on the REACTOME database and at maximum 500 pathways were plotted that were significant after multiple testing.

### Pseudo time analysis

Pseudo time analysis was performed for healthy and for HPV vulva separately. The R-package *monocle3* was used to determine pseudo time. To identify genes that were associated with pseudo time, a linear model was run with participant id as covariate. P-values were adjusted for testing based on the Bejamini-Hochberg procedure and an adjusted P-value below 0.05 was considered significant.

### Neighborhoods and distance between cells

Neighborhoods of cells were identified across individuals. For this, we first identified for each cell in each patient the 30 nearest neighboring cells using the *BuildNicheAssay* function implemented in the Seurat package. Next, we merged the list of Seurat objects to a single object using the *Merge_Seurat_List* function from the Seurat package.

From the merged data, we obtained the scaled sums of cell labels neighboring other cells and, on this data, we performed k-means clustering (20 centers, 30 random sets). Only cell types that represented at least 2% of the niche were shown in plots. Differences in niche frequency between groups were identified based on a Wilcoxon rank test.

The distance between cells was calculated based on the centroids of cells using Pythagoras’ theorem. The cell with the closest centroid distance was considered the closest cell.

### External data sources

Differentially expressed genes were compared to previous datasets that investigated the effects of HPV on epithelial cells. Differentially expressed genes were obtained from published tables. Included studies are listed in **Supplementary Table S5**. Genes were scored for the direction of effect and their significance and expressed as a ratio. The ratio was defined as:


$$\log2(\frac{\%\;studies\;up+1}{\%studies\;down+1})$$

To find VSCC-specific genes, microarray data from Micci et al. were obtained from GSE5884 (20). The R package *limma* was used to run a linear model on expression levels. To compare HPV+ VSCC with HPV- VSCC, RNA-seq data in the form of raw counts aligned by NCBI (GRCh38.p13) was obtained from GSE183454. Using the R package *edgeR*, differentially expressed genes (DEGs) were identified (21). Affymetrix U133 microarray data of HD-1^bri^ and HD-1^dim^ cells were obtained from GSE17014 (22). The R package *limma* was used to run a linear model on expression levels. The log-transformed fold change was averaged across probes for each gene and compared to the fold changes observed in the current study. Data from Love et al. (23) was obtained and annotated using the pipeline described above. Differentially expressed genes for vulvar lichen sclerosis lesions were obtained from Sun et al. (24). For the psoriasis data from Swindell et al. (25) and the Epstein-Barr-virus data from Singh et al. (26) counts were downloaded and DEGs were identified using the glmQLFit function from the R-package edgeR. Plots were produced using the in-house R package CosMXplorer.

### Statistical analysis and figures

All analyses were performed in R 4.5.0. Graphs were produced with *ggplot2*. Dot plots were made using the *DotPlot* function from the R package *Seurat*. Enrichment network plots were made using the *emapplot* function implemented in the *enrichplot* R package. Percentages of main cell types were defined as the number of cells of that type compared to all cells detected in a patient. Percentages of cell subtypes were defined as the number of cells of a subtype within a patient relative to all cells of the parent cell type. Differences in percentages of subclusters between healthy and vHSIL were tested using a Wilcoxon signed-rank test, and a P-value below 0.05 was considered significant.

DEGs between main cell types were defined based on the Wilcoxon rank sum test, and an adjusted P-value below 0.05 was considered significant.

## Results

### Spatial transcriptomics tracks healthy vulvar epithelial differentiation

**Figure 1:**
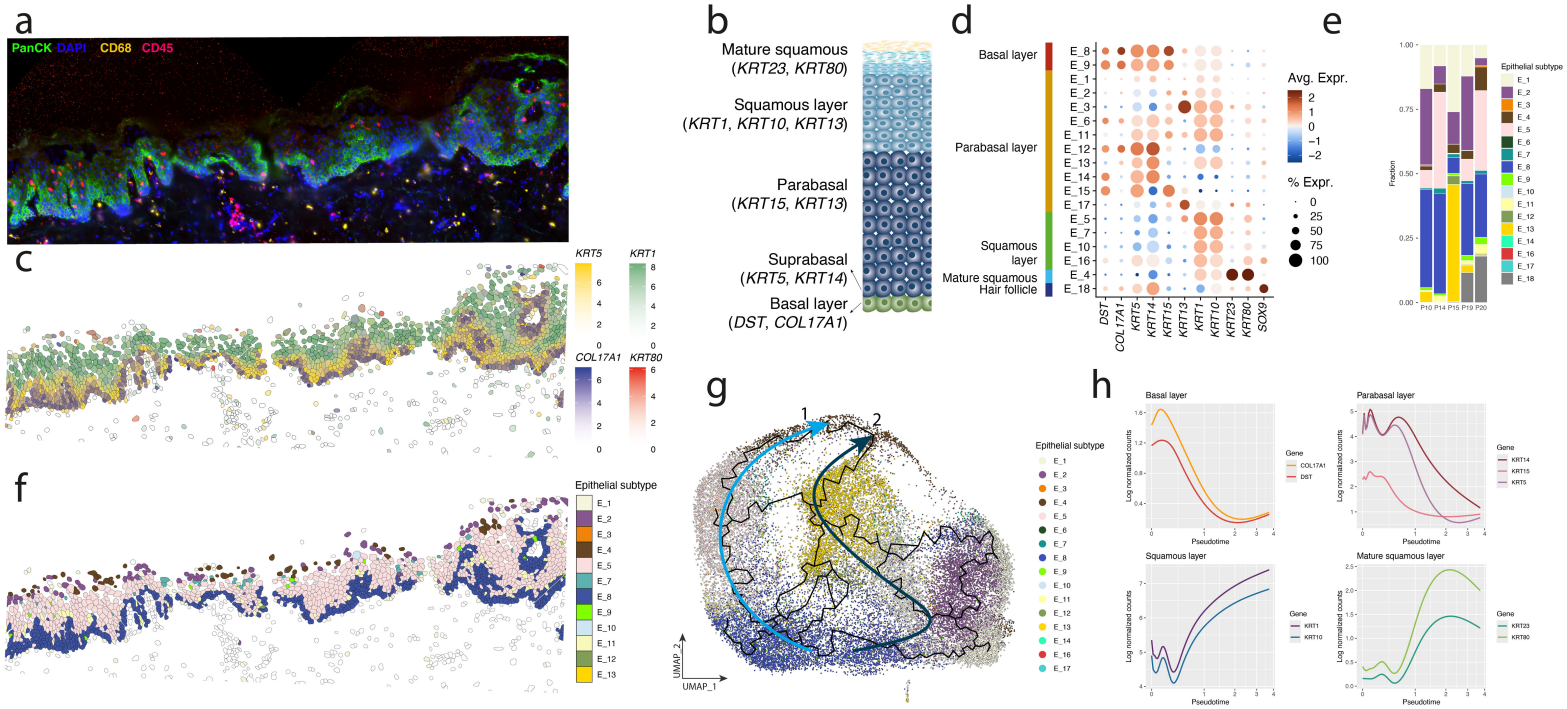
Epithelial differentiation in healthy vulva. **(a)** Immunofluorescence staining of healthy vulvar epithelium. **(b)** Markers of normal differentiating epithelium. **(c)** Key markers for epithelial differentiation plotted on top of each other in an example of healthy vulva **(d)** Comparison of key epithelial differentiation markers in the identified epithelial subtypes in healthy and HPV epithelium. **(e)** Fractions of epithelial subtypes in healthy vulva. Horizontal-axis, individuals; vertical-axis fraction of epithelial cells within total number of epithelial cells. **(f)** Example of spatial location of epithelial subtypes in healthy vulva. **(g)** Pseudo time trajectories of epithelial differentiation in healthy vulvar tissue. Different colors indicate different epithelial subtypes. Arrows indicate the two different identified trajectories in healthy epithelium. **(h)** Expression levels of key genes versus pseudo time.

Single-cell spatial gene expression profiles were generated from healthy (n=5) and HPV16-induced vHSIL (n=31) FFPE sections pre- and post-experimental vaccination against HPV16E6 and E7 (20 pre-vaccination samples, 11 paired post-vaccination samples, **Supplementary Table S1**). In total 415,461 cells were analyzed, of which 285,820 epithelial cells. Major cell types were identified through their canonical markers (**Figure 1a**; **Supplementary Figures S1a, b**) (27). Epithelial cell differentiation was tracked in detail, showing that key markers were expressed as expected within the healthy vulva. *COL17A1* was expressed in the basal membrane, *KRT5* in the parabasal layer, *KRT1* in the squamous layer, and *KRT80* in the mature squamous layer (**Figures 1b, c**). All epithelial cells detected in vHSIL and healthy vulvar tissue were subclustered and grouped based on the differentially expressed genes and their respective location in the epithelium (**Figure 1d**). This resulted in two basal subtypes, ten parabasal subtypes, four squamous layer subtypes, one mature squamous subtype, and one subtype that did not have canonical epithelial markers. Upon further inspection, the latter cells were found in hair follicles and marked by *SOX9* (**Supplementary Figures S1c-e**) (28). For healthy vulva, the most frequent epithelial subtypes were epithelial 8 (E_8), marking the basal membrane, E_5 the squamous layer, and E_4 in the mature squamous layer (**Figures 1d-f**). Frequent parabasal subtypes included E_1, E_2, and E_13. While E_1 and E_2 were present in all samples but not further defined by specific gene expression profiles (**Supplementary Figure S1c**; **Supplementary Table S2**), E_13 was found in a single individual with a slightly aberrant epithelial profile, marked by high *KRT6A/B/C* expression (**Figure 1e**; **Supplementary Figure S1c**). Pseudotime analysis of cell differentiation suggested two different paths of differentiation within healthy vulva, with all differentiation paths ending in the mature squamous layer E_4 (**Figure 1g**). One path showed a differentiation from E_8 to E_5 and ending at E_4, the other started from E_8, and the sequentially followed E_2, E_1, E_13, to end at E_4 (**Figure 1g**). Epithelial differentiation markers followed pseudo time, with high levels of *DST*, *COL17A1* and *KRT5* early in differentiation, followed by high levels of *KRT14* and *KRT13* in the parabasal layer, high levels of *KRT1* and *KRT10* in the squamous layer and finally high levels of *KRT23* and *KRT80* in the mature squamous layer (**Figure 1h**). In addition to these keratins, expression of other genes was positively correlated with pseudo time, including *ARG1*, *CD36* and *GPX3* (**Supplementary Table S3**; **Supplementary Figures S2a-c**). Conversely, for example *CXCL14*, *MT1X*, and *SCGB3A1* were downregulated, and the latter gene is a known basal membrane marker (**Supplementary Figures S2d-f**). Verification of these markers in spatial transcriptomics of human skin from Love et al. (23) showed that *CXCL14* and *ARG1* are not specific to the vulva and are also markers of the basal layer and stratum corneum in the skin, respectively (**Supplementary Figures S2g, h**).

### Inflammation-related genes are upregulated in HPV16-infected epithelium

**Figure 2:**
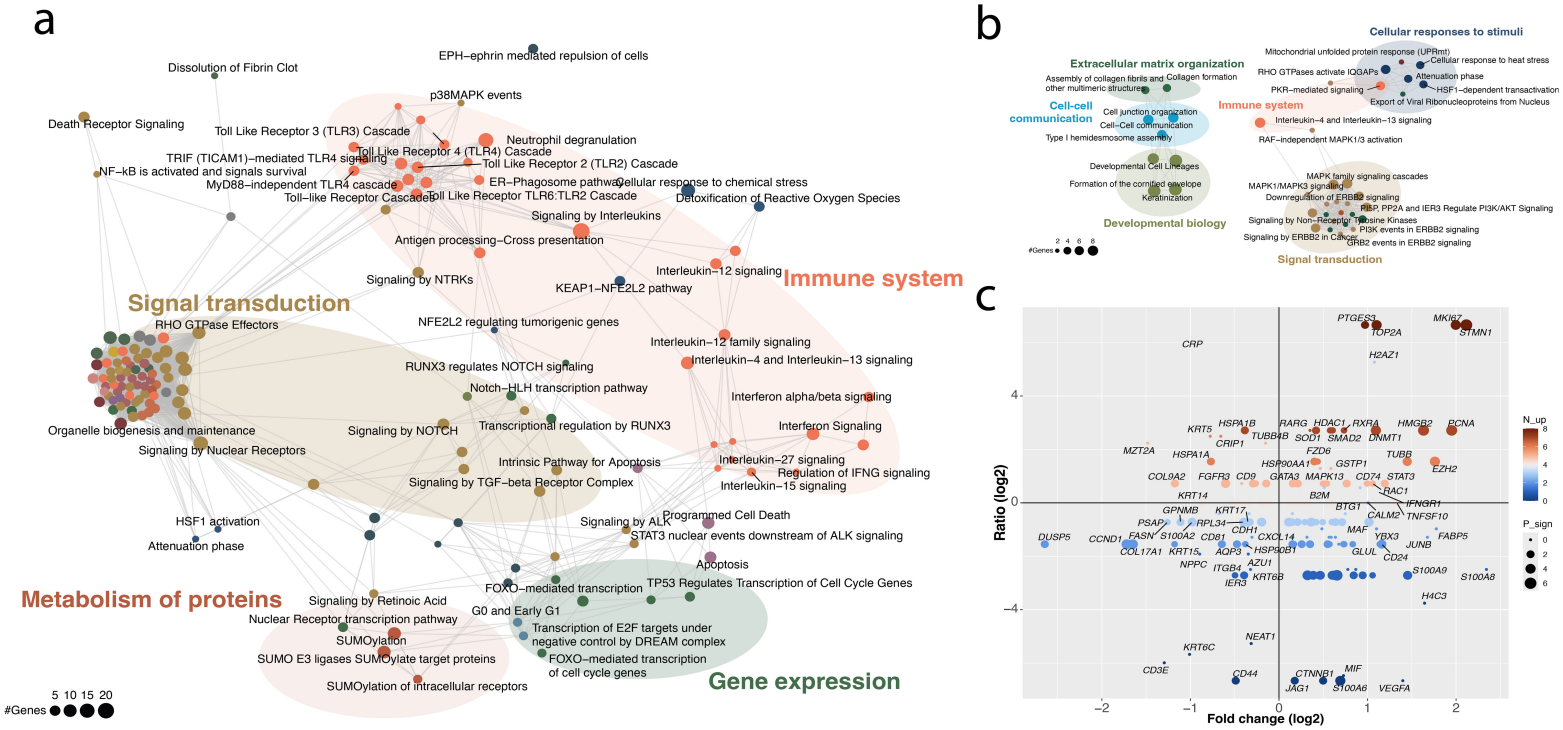
HPV-infected vulvar epithelium is marked by upregulated inflammation and proliferation. **(a, b)** Enriched upregulated **(a)** and downregulated **(b)** pathways in HPV16+ vHSIL epithelium versus healthy vulvar epithelium. **(c)** Differentially expressed genes in the HPV16+ vHSIL epithelial cells versus external *in vitro* studies that investigated the transcriptomic effect of HPV16 on keratinocytes. X-axis, log fold change; y-axis, log2 ratio between the percentage of studies in which the gene was upregulated or downregulated.

On a transcriptional level, healthy and HPV16+vHSIL epithelium displayed 141 differentially expressed genes (40 DEGs down and 101 DEGs up) in the HPV16-infected versus healthy vulvar epithelia (**Supplementary Table S4**). Upregulated genes in vHSIL epithelial cells were primarily involved in the immune system, signal transduction, and metabolism of proteins (**Figure 2a**). MAPK signaling was one of the key altered pathways, including upregulation of upstream alarmins (*S100A8*, *S100A9*), p38 (*MAPK13*, *MAPK14*), AP-1 constituents (*JUNB*), and downregulation of negative regulators of MAPK signaling (*DUSP5*, *HSP72*). Immune system pathways related to signaling by interleukins, interferons, and Toll-like receptors (TLR). Downregulated genes were enriched for pathways involved in, for example, signal transduction, and keratinization (**Figure 2b**).

Although no probes were present to directly detect HPV, a series of HPV-induced DEGs detected *in situ* in vHSIL epithelial cells recapitulated the changes in gene expression commonly found in the data from a compendium of studies investigating the effects of HPV oncogenes on keratinocytes *in vitro* (**Supplementary Table S5**). For example, *DUSP5*, *CCND1, COL17A1*, and *KRT6A/B/C* were downregulated both *in situ* and *in vitro* (**Figure 2c**). Shared upregulated genes were involved in increased proliferation (*STMN1*, *MKI67, TOP2A*, and *PCNA*) and chromatin modification (*EZH2*, *DNMT1*, *HMGB2*, and *HDAC*, **Figure 2c**). To investigate to what extent the observed gene expression differences were specific to HPV infected epithelium, we compared our DEGs to gene expression changes observed in vulvar lichen sclerosis (**Supplementary Figure S3a**), showing that for the great majority of differentially expressed genes in vHSIL did not show the same effect in vulvar lichen sclerosis. The same was observed when the DEGs in HPV16+ keratinocytes were compared to vulvar lichen sclerosis (**Supplementary Figure S3b**). In addition, we compared the DEGS in vHSIL and in HPV16+ keratinocytes versus the inflammatory disease psoriasis, and to infection of keratinocytes with Epstein-Barr-virus (EBV). Again, there was a poor correlation between the DEGs identified (**Supplementary Figures S3c-f**). A few inflammatory genes that were shared with vulvar lichen sclerosis and psoriasis were *S100A8, S100A9* and *FABP5* (**Supplementary Figure S3**). This indicates that the signature we observed is specific for HPV.

**Figure 3:**
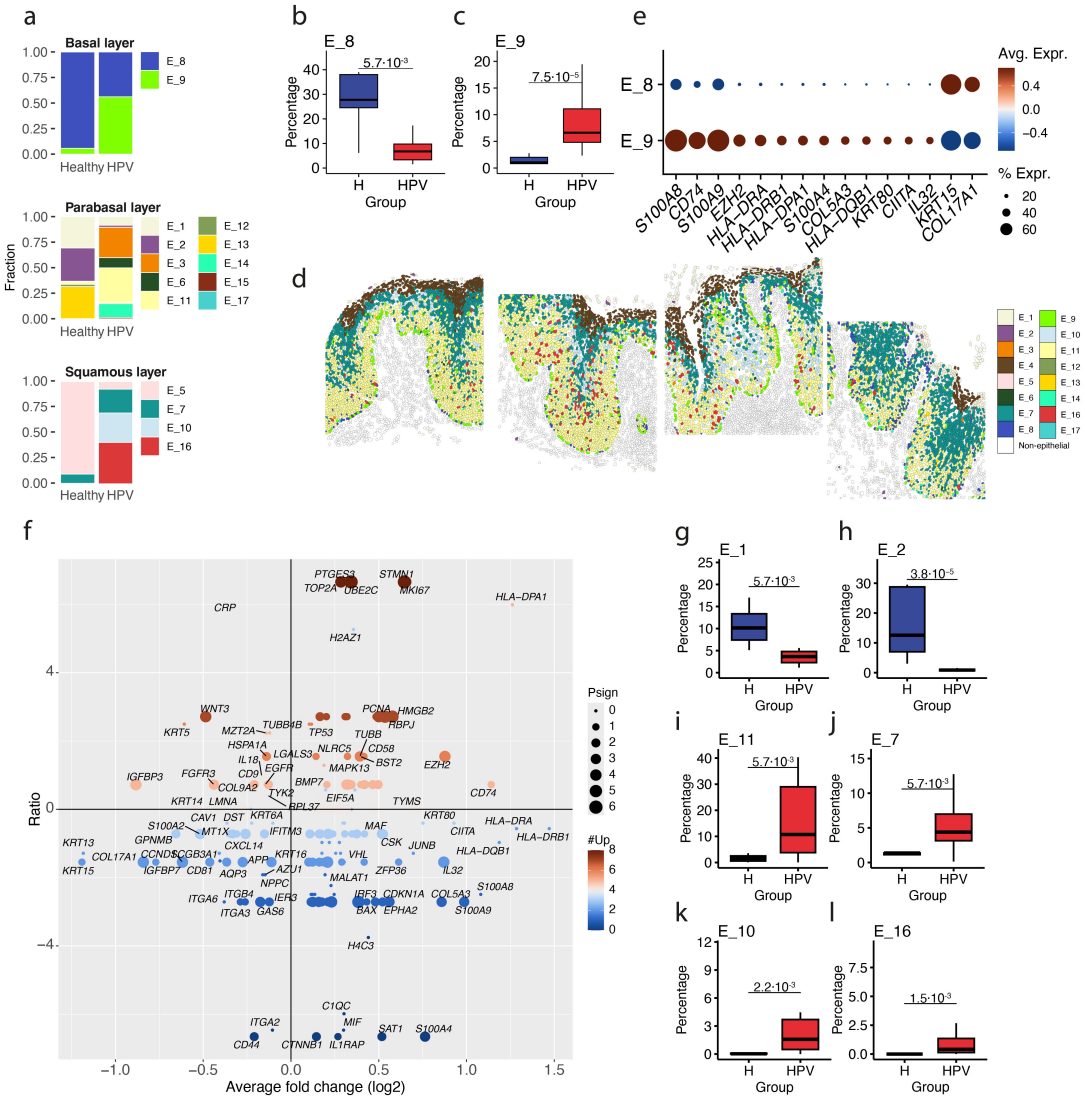
Epithelial subtypes in HPV-infected epithelium. **(a)** Fractions of epithelial subtypes in healthy vulvar (healthy) and vHSIL epithelium (HPV) shown for the basal layer, the parabasal layer and squamous layer. **(b, c)** Percentage E_8 **(b)** and E_9 **(c)** in healthy vulvar (H) and vHSIL epithelium (HPV). **(d)** Example of epithelial subtypes within the spatial context in one HPV16+ vHSIL specimen. Different panels represent adjacent fields of view of the epithelium. **(e)** Differentially expressed genes between E_8 and E_9. **(f)** Differentially expressed genes between E_8 and E_9 versus previously identified DEGs in *in vitro* studies that investigated the effect of HPV16 on keratinocytes. **(g–l)** Percentages of E_1 **(g)**, E_2 **(h)**, E_11 **(i)**, E_7 **(j)**, E_10 **(k)** and E_16 **(l)** in healthy vulvar (H) and vHSIL epithelium (HPV). Significance based on a Wilcoxon signed-rank test.

Notably, the transcriptional differences observed between healthy and vHSIL epithelium were also captured in the epithelial subtypes. Specifically, two basal subtypes were identified, E_8 and E_9, with E_9 being the dominant basal cell subtype in vHSIL (P = 7.5·10^-5^, **Figure 3a-c**). Indeed, in several patients the basal membrane was comprised of almost exclusively E_9 subtype basal cells (**Figure 3d**; **Supplementary Figures S4a-c**). Based on the transcriptional differences, E_9 was the inflamed counterpart of E_8, with upregulation of alarmins *S100A8* and *S100A9* and the increased expression of multiple MHC class II genes (**Figure 3e**). Of note, the upregulation of *S100A8* and *S100A9* was not limited to the basal membrane and was seen throughout the HPV16-changed epithelium (**Supplementary Table S4**; **Supplementary Figures S4d, e**). A comparison of the DEGs expressed between the more abundant in healthy vulvar tissue basal cells E_8 and the HPV-associated basal cells E_9 (**Figure 3a**), revealed that the E_9 basal cells displayed a transcriptome that was highly similar to that of the *in vitro* HPV16 infected keratinocytes (**Figure 3f**; **Supplementary Table S5**), suggesting that E_9 basal cells reflect HPV16-infected cells.

In the parabasal layer, E_1, and E_2 were more frequent in healthy epithelium whereas E_11 was more frequent in vHSIL epithelium (**Figure 3g-i**). In line with this, the DEGs characterizing E_11 showed a consistent effect size to those previously shown to represent HPV16 infection of keratinocytes *in vitro* (**Supplementary Table S5**; **Supplementary Figure S5**). In the squamous layer, E_7, E_10, and E_16 were more frequent in HPV epithelium (**Figure 3j-l**). In line with the effects of HPV, the E_16 and to some extent E_10 and E_11 cells displayed upregulation of genes associated with proliferation such as *STMN1* and *PCNA*. E_16 also showed higher expression of genes from the Wnt pathway, including the receptor Frizzled-6 (*FZD6*) and its ligand *WNT2B*, and the downstream genes, β-catenin (*CTNNB1*) and *STAT3*, but also the Wnt antagonist Wise (*SOSTDC1*). Furthermore, similarly to E_9 and E_11, transcriptional changes in E_16 overlapped with the effects of HPV on the transcriptome of keratinocytes cultured *in vitro* (**Supplementary Figure S5**). For the mature squamous layer, no different subtypes were observed between healthy and vHSIL epithelium. Of note, DEGs shown to represent HPV infection that differ in effect size between the different clusters of epithelial cells may reflect differences in viral DNA levels and/or transcriptional activity.

In a recent study of HPV-infected keratinocytes, a population of epithelial cells was identified that may support carcinogenesis of cervical cancer (5). Similarly, we compared the DEGs existing between healthy vulvar cells versus vulvar squamous cell carcinoma (VSCC; VSCC-signature) and the DEGs between HPV- and HPV+ VSCC (20, 21) to our epithelial subtypes, of which cells that already displayed an HPV16 signature scored higher for the VSCC signature (E_9, E_10, E_16, **Supplementary Figure S6**), but also higher for the HPV+ VSCC signature versus HPV-negative VSCC. Hence, there was no specific subpopulation potentially underlying VSCC development.

### HPV16-infected epithelium results in adaptive immunity supporting changes in different types of cells in the underlying stroma

**Figure 4:**
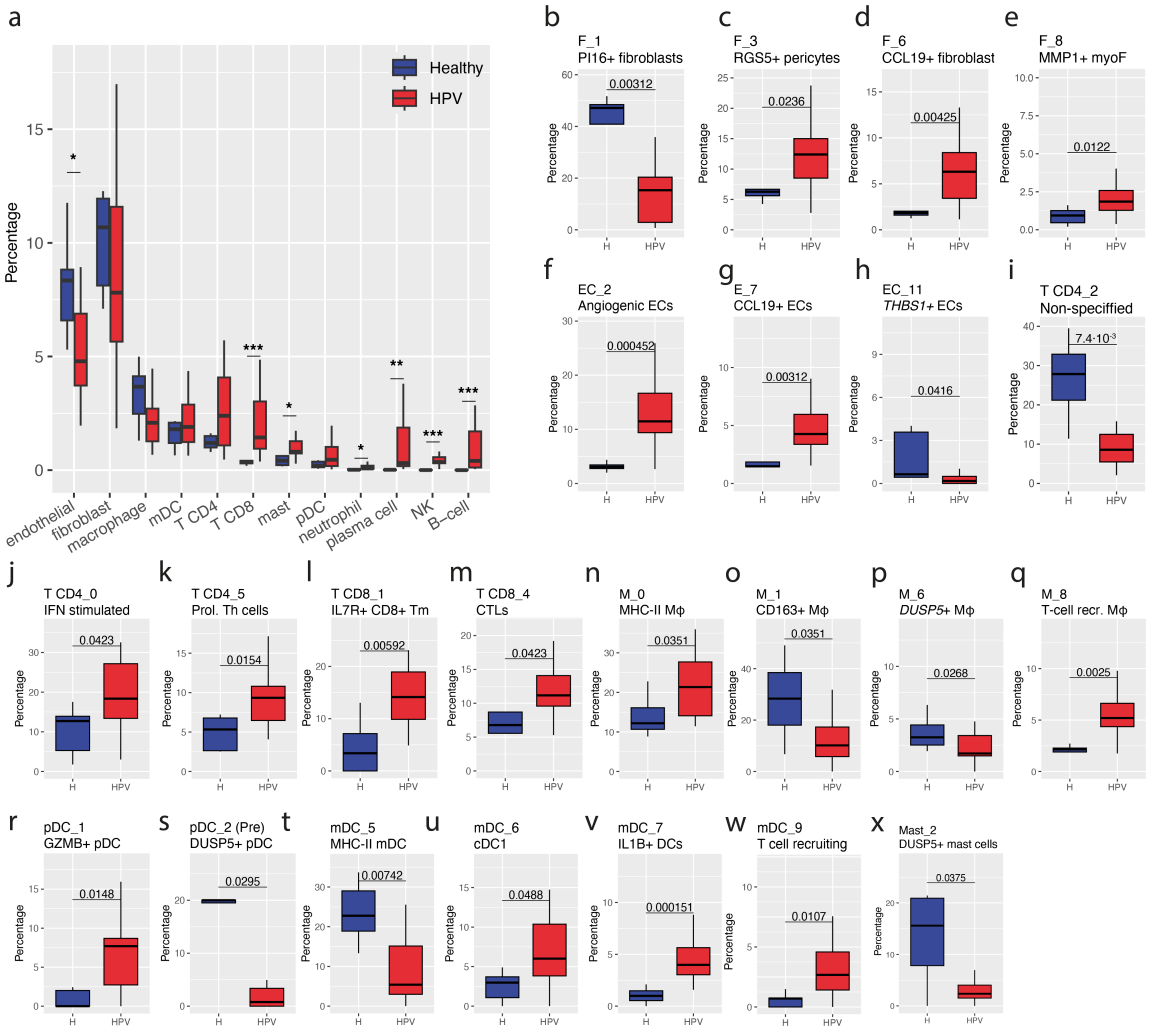
Percentages of cell subtypes in healthy vulvar and HPV-infected vulva. **(a)** Percentages of main cell types in healthy vulvar (healthy) versus vHSIL epithelium (HPV).**(b–x)** Fractions of cell subtypes for fibroblasts **(b–e)**, endothelial cells **(f–h)**, CD4+ T cells **(i–k)**, CD8+ T cells **(l–m)**, macrophages (n-q), pDCs (r-s), mDCs **(t–w)** and mast cells **(x)** in healthy vulvar (H) and vHSIL epithelium (HPV). X-axis, group; y-axis percentage of cell subtype out of the total number of parent cells. Significance based on a Wilcoxon rank test.

Provided the changes in the epithelium, we next investigated the effects of HPV16 infection on the stromal cells. In line with the increased expression of immune-related genes by HPV16-infected epithelia, we observed a general higher immune infiltrate in vHSIL than normal vulvar tissue. CD8+ T cells (P = 6.1·10^-5^), NK cells (P = 2.9·10^-4^), B-cells (P = 2.9·10^-4^), plasma cells (P = 1.4·10^-3^) and mast cells (P = 0.03) were enriched in vHSIL versus healthy vulvar tissue (**Figure 4a**). Although CD4+ T cells were also more frequent in some patients, this was not significantly different (P = 0.11). Furthermore, a decreased percentage of endothelial cells (P = 0.03) was observed (**Figure 4a**).

To perform a more in-depth analysis, the main cell types were subclustered and annotated (**Supplementary Table S6**). The fibroblast population comprised 10 subpopulations. F_1 fibroblasts were more prominent in healthy vulvar tissue and reflect the PI16+ progenitor-like fibroblasts, more often detected in healthy tissue (13). In contrast, several fibroblast subpopulations, F_3 (P = 0.02), F_6 (P = 0.004), and F_8 (P = 0.01), were present at higher frequencies in vHSIL (**Figures 4b-e**). The F_3 subpopulation closely resembled RGS5+ pericytes, suggested to play a role in tissue skin regeneration (22). This was further supported by a good correlation (r=0.61, P = 2.7·10^-43^) between F_3 DEGs and previously identified DEGs in pericytes from human neonatal foreskin (**Supplementary Figure S7a**; **Supplementary Table S5**), including *RGS5*, *TAGLN*, *ACTA2* and *NOTCH3* (**Supplementary Table S6**) (22). The F_6 CCL19+ fibroblasts have been shown to interact with T-cells via CXCL12/CXCR4 and F_8 MMP1+ myofibroblasts (myoF) have recently been linked to an immunosuppressive neighborhood in cancer (13, 29, 30). The endothelial cell population comprised 12 subclusters. Whereas the percentage of thrombospondin-1+ (*THBS1*) ECs (EC_11) were higher in healthy vulvar tissue, two other subtypes (EC_2 & EC_7) were increased in vHSIL (**Figures 4f-h**). EC_2 was marked by upregulation of genes involved in angiogenesis, including the vascular endothelial growth factor receptor 1 and 2 (*FLT1*, *KDR*) and endoglin (ENG). In addition, the insulin receptor was upregulated, which has shown to play a role in the response to VEGFA via the VEGFR2 (31). EC_7 are CCL19+ venous ECs, where *CCL19* has been shown to have a promigratory role via *CCR7* and *CCRL1* (31, 32).

For the immune cells, non-specified T cells (T CD4_2, P = 7.4·10^-3^, **Figure 4i**) were less present in vHSIL stroma, while homeotypic cytokine producing of IFN-stimulated T cells (T CD4_0, P = 0.04) and proliferating T helper cells (T CD4_5, P = 0.02) increased in frequency as part of the ten CD4+ T cell populations in vHSIL (**Figures 4j, k**). Out of the nine CD8+ T cell populations, an increased presence was observed for IL7R+ CD8 memory T cells (T CD8_1, P = 5.9·10^-3^) and activated cytotoxic terminally differentiated tissue resident T cells (T CD8_4, P = 0.04, **Figures 4l, m**). IL7R+ CD8 T cells have been shown to be a stem-like tumor-reactive CD8 T cell population as was shown in head and neck cancer (Eberhardt et al., 2021) and is related to a better clinical outcome upon immunotherapy (33, 34).

Out of the eleven macrophage subtypes, CD163+ M2c (M_1, P = 0.03) and DUSP5+ macrophages (M_6, P = 0.03) were less frequent in HPV stroma, while MHC class II-low non-activated (M_0, P = 0.04) and T-cell recruiting macrophages (M_8, P = 2.5·10^-3^) were more present (**Figures 4n-q**). For the dendritic cells, we identified six plasmacytoid dendritic cell (pDC) subtypes and ten monocytic dendritic cell (mDC) subtypes. In vHSIL stroma, a lower frequency of DUSP5+ pDCs (pDC_2, P = 0.03) was observed, while GZMB+ IFN activated pDCs were more frequent (pDC_1, P = 0.01, **Figures 4r, s**). Similarly, MHC class-II high mDC were downregulated in vHSIL (mDC_5, P = 7.4·10^-3^), while IL1B+ DCs (mDC_7, P = 1.51·10^-4^), cDC1s (mDC_6, P = 0.05) and T cell recruiting DCs (mDC_9, P = 0.01) were more present in vHSIL stroma (**Figures 4t-w**). Because myeloid derived suppressor cells (MDSCs) are known to be immune suppressive, we specifically analyzed the presence of these cells, yet they were only sporadically found (**Supplementary Figure S7b**).

For mast cells we identified three specific subtypes, one of which was lower in vHSIL stroma (P = 0.04) and marked by higher *DUSP5* expression (**Figure 4x**). The DUSP5+ subpopulations of macrophages, mast and pDCs, which were more prominent in healthy vulvar tissue, are likely to play an anti-inflammatory role (35–37). While a general increase in B-cells and NK cells frequency was observed in vHSIL, no specific B-cell subtypes specifically stood out (P>0.05) and NK cell subclustering did not result in distinct clusters. Neutrophils were found in the different types of tissue, but no differences were observed when analyzed as a group or as subtypes.

Overall, it was clear that the immune activation observed in HPV16-infected epithelial cells had an impact on several different stromal cells, including fibroblasts, endothelial cells and different types of immune cells, responding to this immune activation signal.

### Neighborhoods of different immune cells form spatial barriers for adaptive immunity in HPV16+ vHSIL

**Figure 5:**
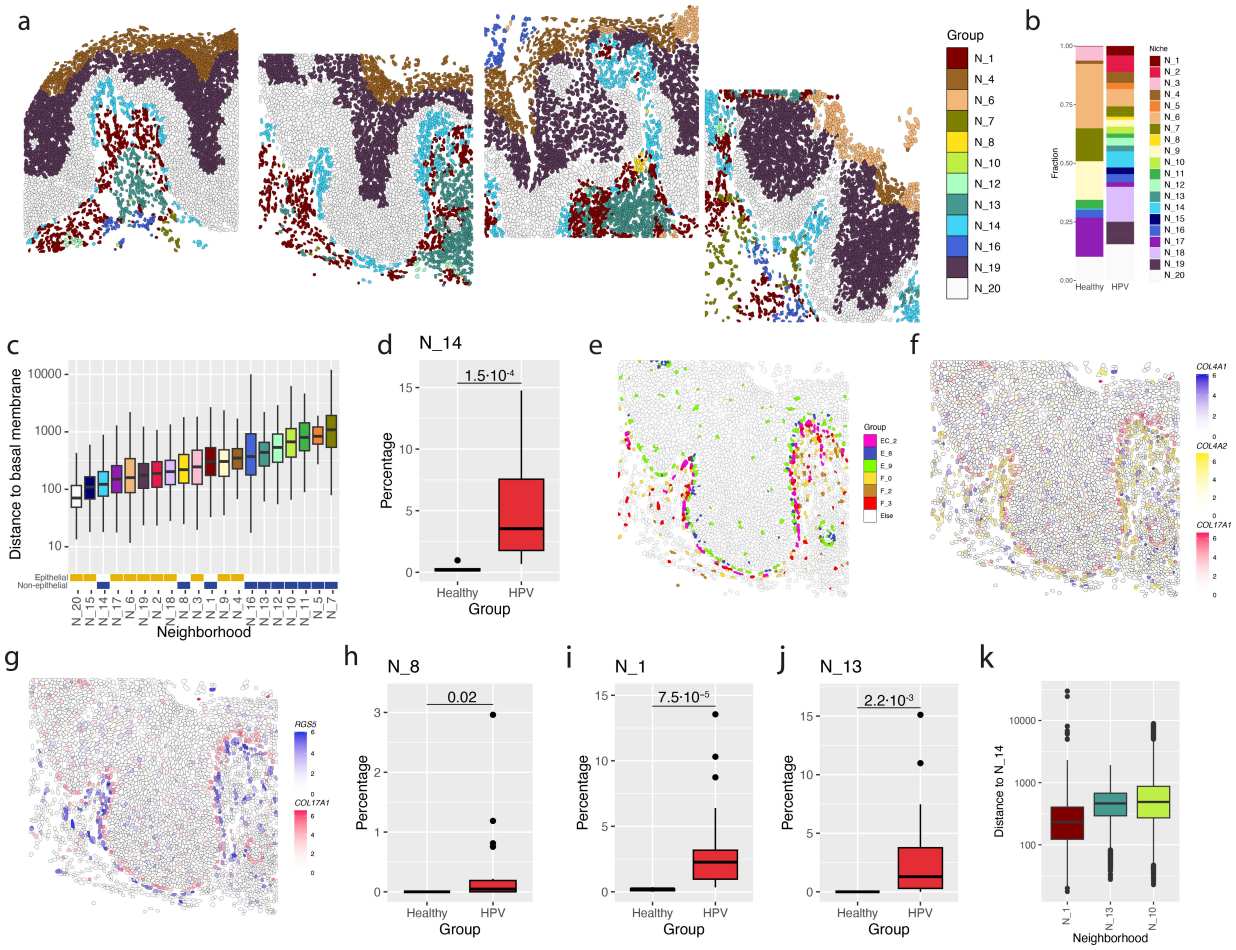
Specific cellular neighborhoods near the basement membrane of HPV-infected epithelium. **(a)** Identified neighborhoods (N) in the spatial context in HPV16+ epithelium. Different panels represent 4 adjacent fields of view in the vHSIL epithelium. **(b)** Frequency of neighborhoods in healthy vulvar (healthy) and HPV16+ vHSIL tissue (HPV). **(c)** Distance between basal membrane and cells within a specific neighborhood. **(d)** Percentage of cells belonging to neighborhood 14 in healthy vulvar (healthy) and HPV16+ vHSIL tissue (HPV). **(e)** Most frequent cell types in neighborhood 14 (N_14) plotted for a single field of view from the HPV16+ vHSIL shown in panel c. **(f)** Expression of *COL4A1*, *COL4A2* and *COL17A1* in a single field of view of HPV16+ epithelium. **(g)** Expression of *RGS5* and *COL17A1* in a single field of view of HPV16+ epithelium. **(h–j)** Percentage of cells in healthy vulvar (healthy) and HPV16+ vHSIL tissue (HPV) belonging to **(h)** neighborhood 8 (N_8), **(i)** neighborhood 1 (N_1) and **(j)** neighborhood 13 (N_13). **(k)** Distance from neighborhood N_14 for neighborhoods N_1 and N_13.

To investigate potential interactions of cell types in healthy and HPV-infected vulvar tissue, specific neighborhoods of different cell subtypes were identified (**Supplementary Figures S8a-t**). Twenty neighborhoods were considered to provide relevant combinations of cell types. Out of the 20 neighborhoods, nine neighborhoods were comprised of solely epithelial cells, neighborhood 3 marking a hair follicle neighborhood, and the others represented a combination of cell types (**Supplementary Figure S8**). For the epithelium, neighborhoods 17 and 20 marked the basal membrane and the squamous layer. For neighborhood 17, this was E_5 combined with basal membrane subtypes E_8, while for neighborhood 20 this was E_11 with basal membrane subtypes E_8 and E_9 (**Figure 5a**; **Supplementary Figure S8t**). Neighborhood 19 represented the squamous epithelium with E_10, E_7 and to a lesser extent E_11. Neighborhood 4 marked the mature squamous layer with E_4 and neighborhood 6 was also found near the mature squamous layer marked by E_2 and E_1. Finally, neighborhoods 2, 9, and 15 were primarily driven by specific epithelial cells (**Supplementary Figure S8**).

In healthy vulva, frequently observed neighborhoods were those encompassing epithelial cells, with the exception of neighborhood 7, which was comprised of many smaller cell fractions, including PI16+ progenitor-like fibroblasts (F_1) and systemic-venous ECs (EC_0, **Figure 5b**). We next focused on the neighborhoods close to the basal membrane as these may interact with the initial HPV16-infected basal cells. Neighborhood 20 was closest to the basal membrane and was comprised of the two basal cell types E_8 & E_9 and the parabasal subtype E_11 (**Figure 5c**; **Supplementary Figure S8**). For the non-epithelial neighborhoods, neighborhood 1, 8, 10, 13, 14 showed a significant difference in frequency between healthy and HPV16+ patients. Of those, neighborhood 14 was distributed along the entire basal membrane, essentially forming it (**Figure 5a, d**, **Supplementary Figures S8n**, **S9**, **S10**). It comprised the two basal cell subtypes E_8 and E_9 and in addition RGS5+ pericytes (F_3), the immunosuppressive LRRC15+ myoF (F_0) and the EC_2, angiogenic endothelial cell (**Figure 5e**; **Supplementary Figure S8n**). Of note, *RGS5* expression was very specific to the cells close to the basal membrane (**Figure 5e**). Based on these cell types, neighborhood 14 may reflect a wound-healing process in which a combination of cells is aiming to repair the basal membrane and suppress immunity in areas of HPV16-altered basal cells (E_9). RGS5+ pericytes have been suggested to play a role in epithelial regeneration in an angiogenesis independent way (22). This was further supported by high expression of collagen IV and RGS5 of cells neighboring the basal membrane, the main constituent of the lamina densa (22, 38), as both EC_2 and F_3 showed upregulation (3.8-5.5x) of genes encoding collagen IV, i.e. *COL4A1* and *COL4A2* (**Figures 5f, g**). Neighborhood 8 was also close to the basal membrane (**Figures 5a, h**), concentrated in single spots (**Figure 5a**; **Supplementary Figure S10**) and comprised several epithelia subtypes (E_13, E_1, E_2), neutrophils (N_0, N_1, N_2) and fibroblasts (F_2, F_9). F_2 are CD74+ antigen-presenting fibroblasts and F_9 are IL6+ fibroblasts that express *CXCL1/2/3*, chemokines that are known to recruit neutrophils (39).

A bit more distal to the basal membrane are neighborhoods 1 and 13, two neighborhoods that were also predominantly found in vHSIL (**Figures 5i, j**). Neighborhood 1 (**Supplementary Figure S8a**) comprised LRRC15+ myoFs (F_0) and RGS5+ pericytes (F_3), together with CD74+ antigen presenting fibroblasts (F_2), systemic-venous ECs (EC_0), undefined mDC (mDC_0) and regulatory T-cells (T CD4_3). The role of mDC_0 cells could not be derived from its DEGs but at least did not mark them as any of the adaptive immunity supporting mDCs (e.g. mDC_1, 3, 6, 8, 9). Neighborhood 13 clearly constituted a hotspot of adaptive immunity. It was populated with lymphatic endothelial cells (EC_10), the earlier mentioned T cell attracting CCL19+ fibroblasts (F_6) and IFN-activated homeostatic cytokine producing CD4 T cells (CD4_0) and also included proliferating CD4 T cells (CD4_5), type 1 effector CD4 T cells (CD4_7), CCL5-producing CD8 T cells (CD8_0) and IL7R+ CD8 memory T cells (CD8_1). In addition, it contained two populations of regulatory T cells (CD4_3 & CD4_4) as well as the F_2 antigen presenting fibroblasts known to stimulate these cells. The increased presence of neighborhood 13 in HVP16+ vHSIL clearly shows the attempt of the immune system to deal with this viral threat. Interestingly, neighborhood 1 is spatially located directly between neighborhoods 14 and 13 (**Figures 5a, k**; **Supplementary Figure S9**), suggesting that this immune suppressive neighborhood may form a barrier that shields off the repair processes in neighborhood 14.

**Figure 6:**
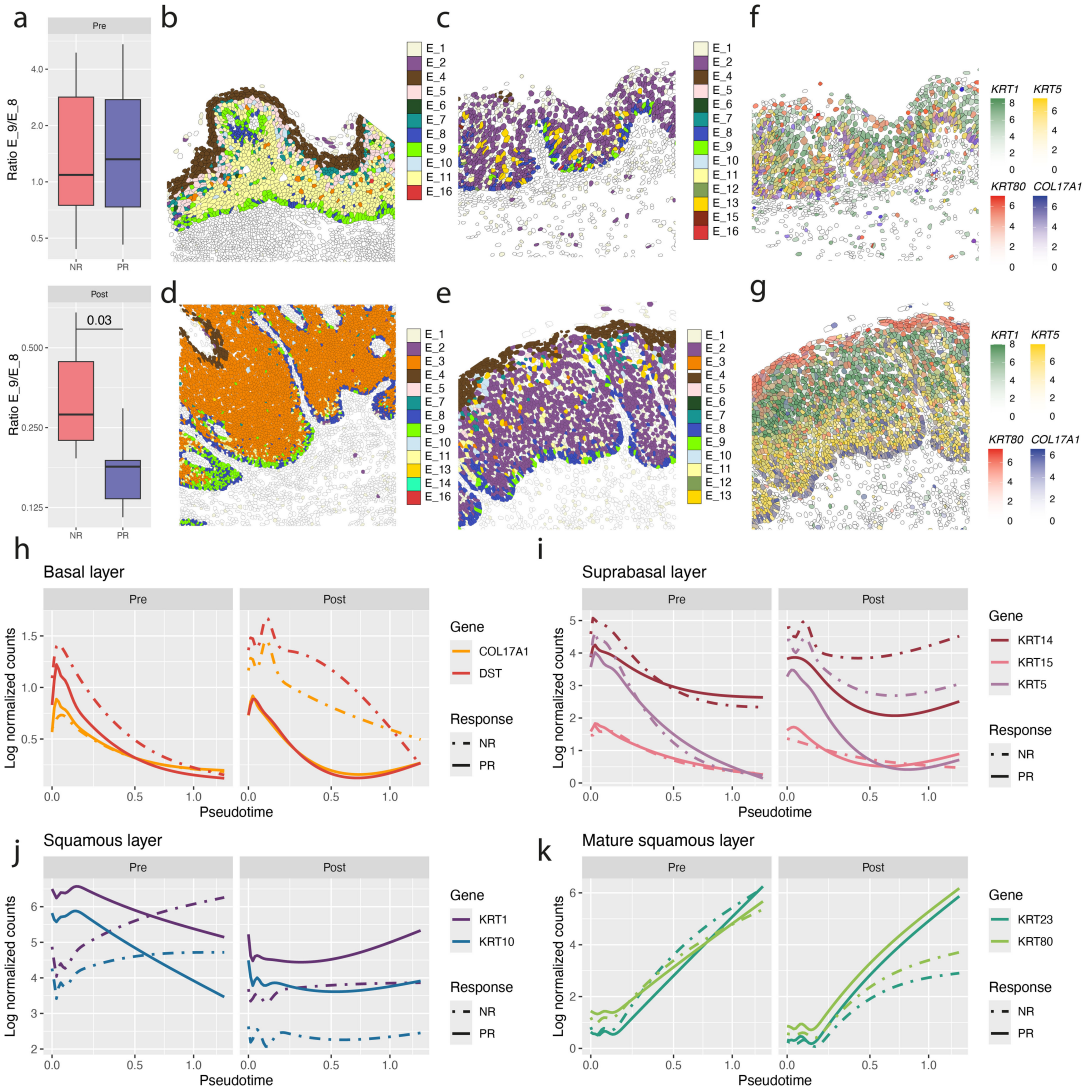
Normalization of basal layer in partial-responders after vaccination. **(a)** Ratio of the HPV-infected E_9 basal cells relative to E_8 basal cells in vulvar tissue of non-responding (NR) and partial responding (PR) patients pre- and post-vaccination in. **(b–e)** Example of epithelial subtypes in a NR pre-vaccination **(b)**, in a PR pre-vaccination **(c)**, in a NR post-vaccination **(d)** and in a PR post-vaccination **(e)**. **(f, g)** Expression of normal basal membrane and epithelial differentiation markers in a NR **(f)** and a PR **(g)** to therapeutic vaccination. **(h–k)** Pseudotime versus expression of key markers involved in epithelial differentiation. Basal layer **(h)**, suprabasal **(i)**, squamous layer **(j)** and mature squamous layer markers are shown **(k)**.

For a number of vHSIL patients, we obtained tissue before and after they were vaccinated with a therapeutic HPV16 vaccine (7, 8). Before therapy, there was no difference in the frequency of HPV16-infected E_9 epithelial cells among all basal cells (ratio E_9/E_8) between the group of patients with no response (NR, n=7) or having a partial response (PR, n=13, **Figure 6a**) after vaccination. However, after therapy the E_9/E_8 ratio was lower in PR patients (N = 6) than NR patients (N = 5, P = 0.03, **Figure 6a**). Moreover, in NR the basal membrane was not intact (**Figures 6b, c**), while in PR, the basal membrane was continuous and primarily comprised of E_8 cells (**Figures 6d, e**), suggesting that the clinical response to therapy is associated with restoration of the basal cell layer. This was sustained by irregular expression of normal basal membrane (*COL17A1*, *DST*) and epithelial differentiation markers (*KRT1*, *KRT5*, *KRT14*, *KRT15*, *KRT23*, *KRT80*) in the NR epithelium postvaccination, while in the PR the expression profile more or less resembled that of the healthy epithelium (**Figures 6f-k**; **Figure 1c**). Specifically, in the postvaccination NR epithelium, basal and suprabasal membrane markers remained high, while the expression of mature squamous markers was lower (**Figure 6k**).

## Discussion

We applied single cell spatial transcriptomics to study the effect of HPV16 infection on the vulvar epithelium. Analysis of all the cells in the healthy vulvar tissue was used to create a digital cell atlas with clear identification of the known different epithelial subtypes, their differentiation path, present in the expected spatial location (27). In addition, it provided an in-depth insight into the different (sub)types of cells constituting the normal vulvar stroma. The effect of HPV16 was deduced by comparing the data obtained from HPV16-induced high-grade vulvar lesions to that of healthy vulva. The epithelium of vHSIL was characterized by the presence of more different epithelial subpopulations, some of which displaying a transcriptomic signature that was highly similar to what was seen in keratinocytes either transfected with HPV16 oncogenes or infected with genuine HPV16 virus (2–4, 40–42). HPV16 infects the cells forming the basal cell layer (43) and we identified epithelial cell clusters 8 and 9 as two subpopulations of basal cells in our vulvar tissues. In healthy tissue, E_8 basal cells predominated, whereas in vHSIL, E_9 basal cells became dominant. Notably, this basal cell subpopulation displayed the strongest similarity to the *in vitro* HPV signature and, therefore, is likely to reflect HPV-infected basal cells, now perfectly visualized in their natural context. The lack of a full transcriptomic resemblance to the *in vitro* HPV signature is likely due to the limited set of genes analyzed here as well as due to the facts that a number of studies utilized keratinocytes transfected with HPV16’s oncoproteins only (3, 4, 40–42) or a genuine infection outside the context of the whole tissue (2) as we performed here. HPV16-infected epithelial cells clearly upregulated the expression of genes associated with proliferation – fitting with the disturbed epithelial differentiation detected in vHSIL - and genes involved in the initiation of immune responses – in line with the increased stromal immune infiltrate detected in vHSIL when compared to healthy vulva. A limitation of the current study is that findings could not be verified on the protein level because all available material was used for the current study.

The effects of HPV16 extended beyond the epithelial cells as also different types of stromal cells were affected, resulting in the increased presence of fibroblasts involved in tissue generation (22), immune cell attraction (29, 30) but also immune suppression (LLRC15+ F_0 (13)). Notably, although LRRC15+ myofibroblasts have also been implicated in antiviral and antifibrotic processes, they are specifically known for their link to cancer (13, 44, 45). Furthermore, an increased presence of endothelial cells involved in angiogenesis and an increased influx of immune cells was detected. To understand the potential functional consequences of these HPV16-associated early changes in the vulvar epithelium we analyzed the spatial location of these cells. Cellular neighborhood analyses revealed that many of these cells were organized in specific neighborhoods providing biological context to the development of HPV-induced disease. Three neighborhoods found with higher frequency in HPV-infected vulvar tissue were neighborhoods 1, 13 and 14. Based on the cells present and their transcriptome-derived function neighborhood 14 reflected areas in which the cells potentially attempt to restore the basement membrane. To guard this wound-healing process neighborhood 14 was shielded from the developing adaptive immune response in neighborhood 13, by a barrier of immune suppressive cells found in neighborhood 1. When successful, this should lead to normalization of the epithelial layers. The observation that the frequency of the HPV16-infected E_9 basal cells was relatively lower and the detection of a restored normal expression of epithelial differentiation markers, in the tissue obtained from partial responders but not non-responders after immunotherapy, adds to this notion.

In conclusion, single cell spatial transcriptomic analysis provided biological context governing the disease process of an epithelial infection with HPV16. High numbers of basal cells were visibly affected by HPV16, disturbing the basement membrane. The host’s response seems to be geared to restoration of the basal layer and includes very local suppression of immunity as indicated by the cellular ecosystems formed around or in its direct vicinity, a balance that not only may locally fend of the aroused adaptive immune response but also may influence clinical outcomes after immunotherapeutic approaches.

## Data Availability

The data presented in the study are deposited in the Gene Expression Omnibus, accession number GSE311892, available via https://www.ncbi.nlm.nih.gov/geo/query/acc.cgi?acc=GSE311892.
