# Supplementary material for: The local cellular response to Human Papillomavirus focuses on basal layer restoration as visualized in situ by specific cellular neighborhoods near infected cells
Source: Front Immunol. 2025 Dec 8;16:1728629. doi: 10.3389/fimmu.2025.1728629 (PMC12719499; doi:10.3389/fimmu.2025.1728629)
Supplement: Supplementary file 1 [file DataSheet1.pdf]

Supplemental Figures

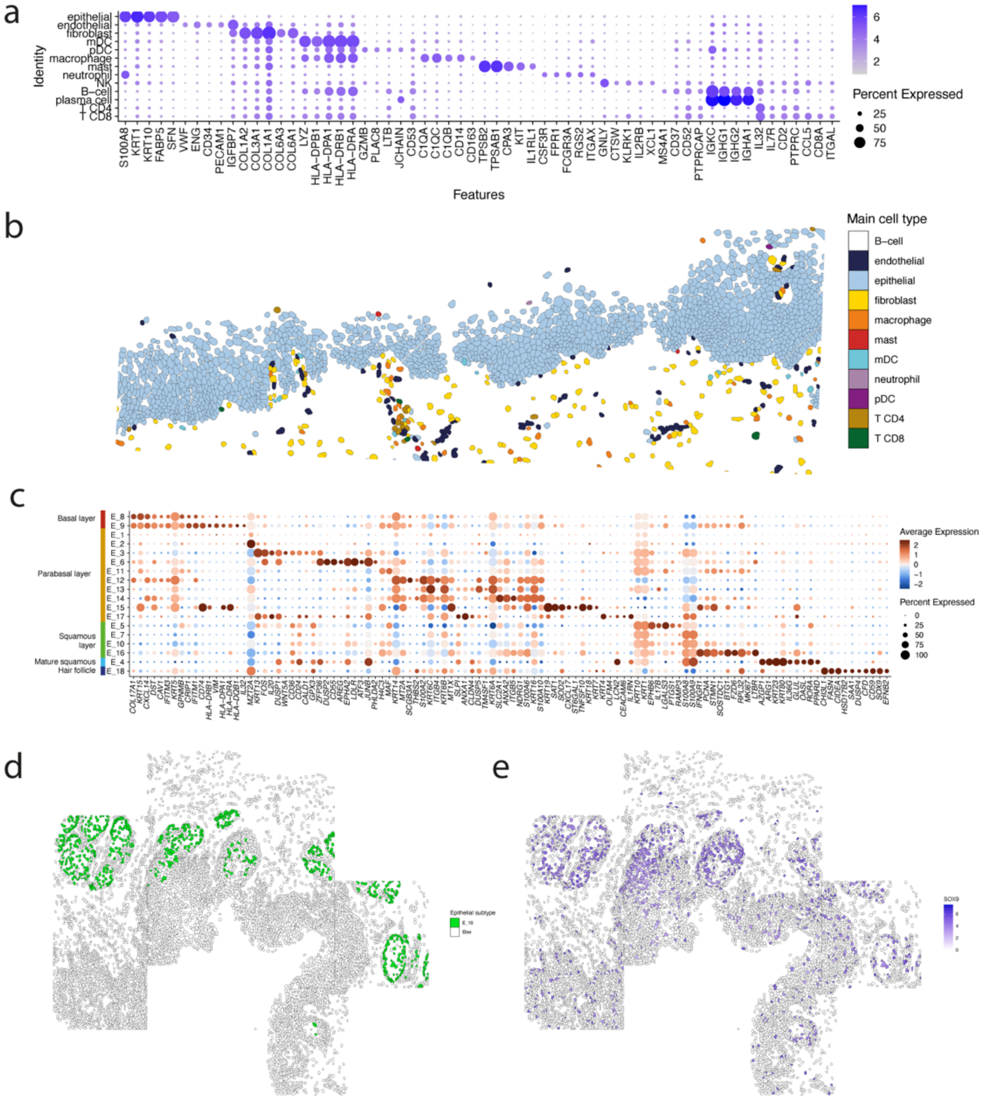

Figure S1. a Differentially expressed genes observed between major cell types. **b** Example plot of main cell types plotted on healthy vulvar tissue. **c** Differentially expressed genes observed between epithelial subtypes. **d,e** Example plot of E_18, representing hair follicle cells (d) as marked by SOX9 (e).

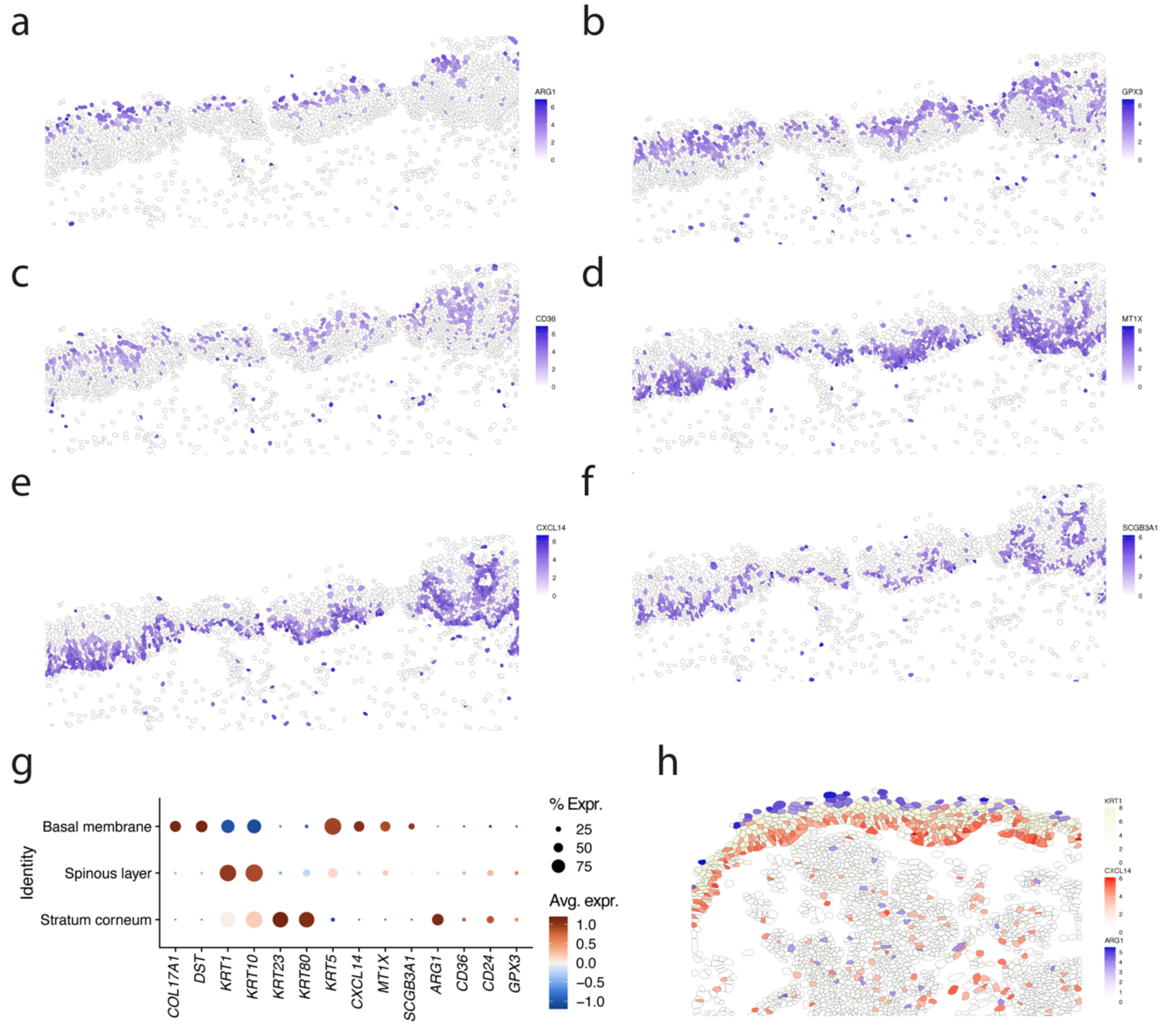

Figure S2. a-f Spatial plot of *ARG1* (a), *GPX3* (b), *CD36* (b), *MT1X* (d), *CXCL14* (e), *SCGB3A1* (f) in healthy vulvar tissue. **g-h** Reanalysis of healthy human skin data from Love et al.[42] with respect to the expression of genes associated with pseudo time in (g) and spatial plot of *ARG1, KRT1,* and *CXCL14* expression (h).

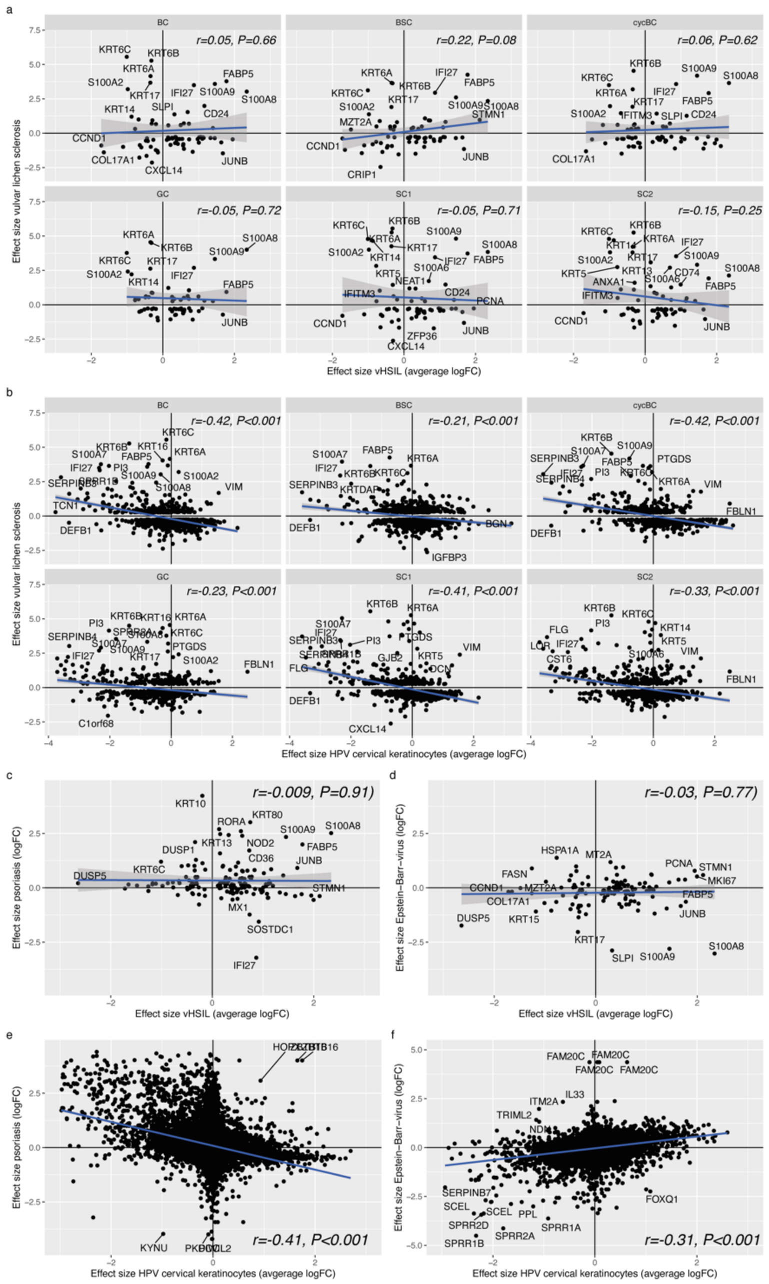

Figure S3. Comparison of DEGs to other pathologies. a-b Comparison of DEGs in the current study (a) and DEGs in HPV16+ keratinocytes (b) versus DEGs identified between lesional and non-lesional vulvar tissue caused by vulvar lichen sclerosis. Correlation values present Pearson's correlation. **c** Comparison of DEGs in the current study to DEGs identified in keratinocytes from patients with and without psoriasis. **d** Comparison of DEGs in the current study to DEGs identified in Epstein-Barr-virus keratinocytes compared to non-infected keratinocytes. **e** Comparison of DEGs in HPV16+ keratinocytes to DEGs identified in keratinocytes from patients with and without psoriasis. **f** Comparison of DEGs in HPV16+ keratinocytes to DEGs identified in Epstein-Barr-virus keratinocytes compared to non-infected keratinocytes.

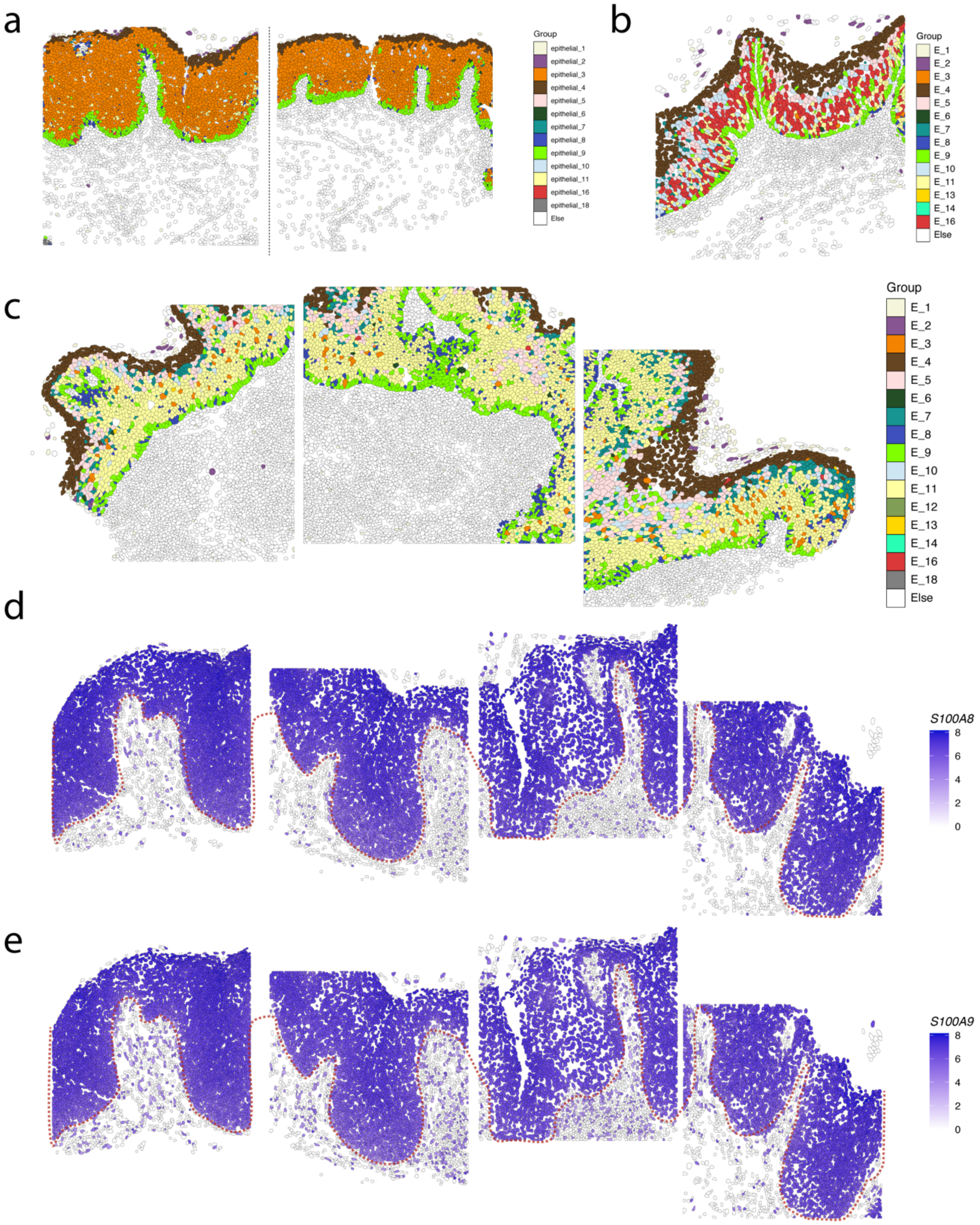

Figure S4. a-c Examples of HPV16 vHSIL epithelium with a high fraction of E_9 cells in the basal layer. **d-e** Spatial expression of S100A8 (d) and S100A9 (e). The red line demarcates the epithelial-stroma junction.

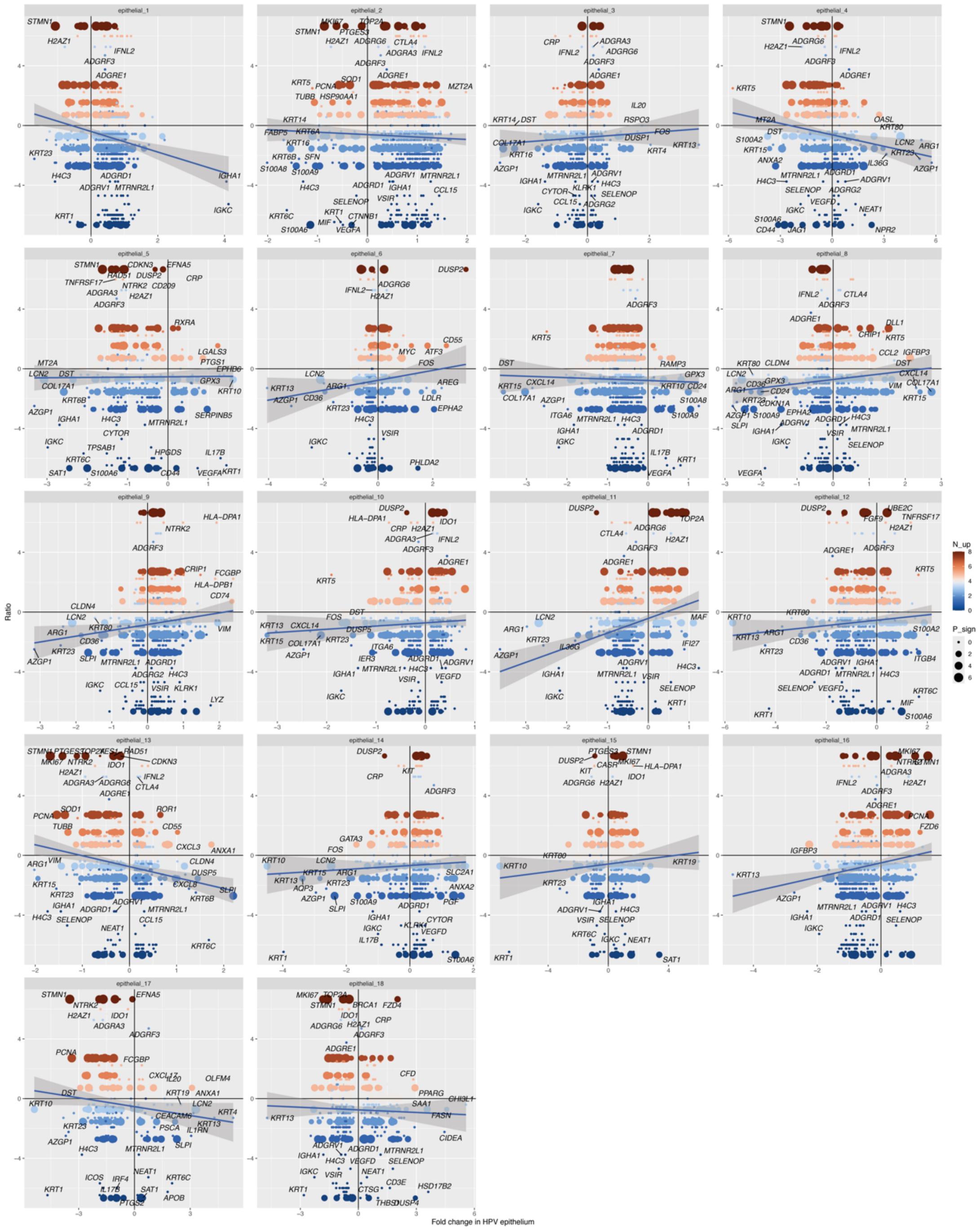

Figure S5. Differentially expressed genes for each epithelial subcluster 1 to 18 versus previously identified DEGs of *in vitro* studies that investigated the effect of HPV16 on keratinocytes.

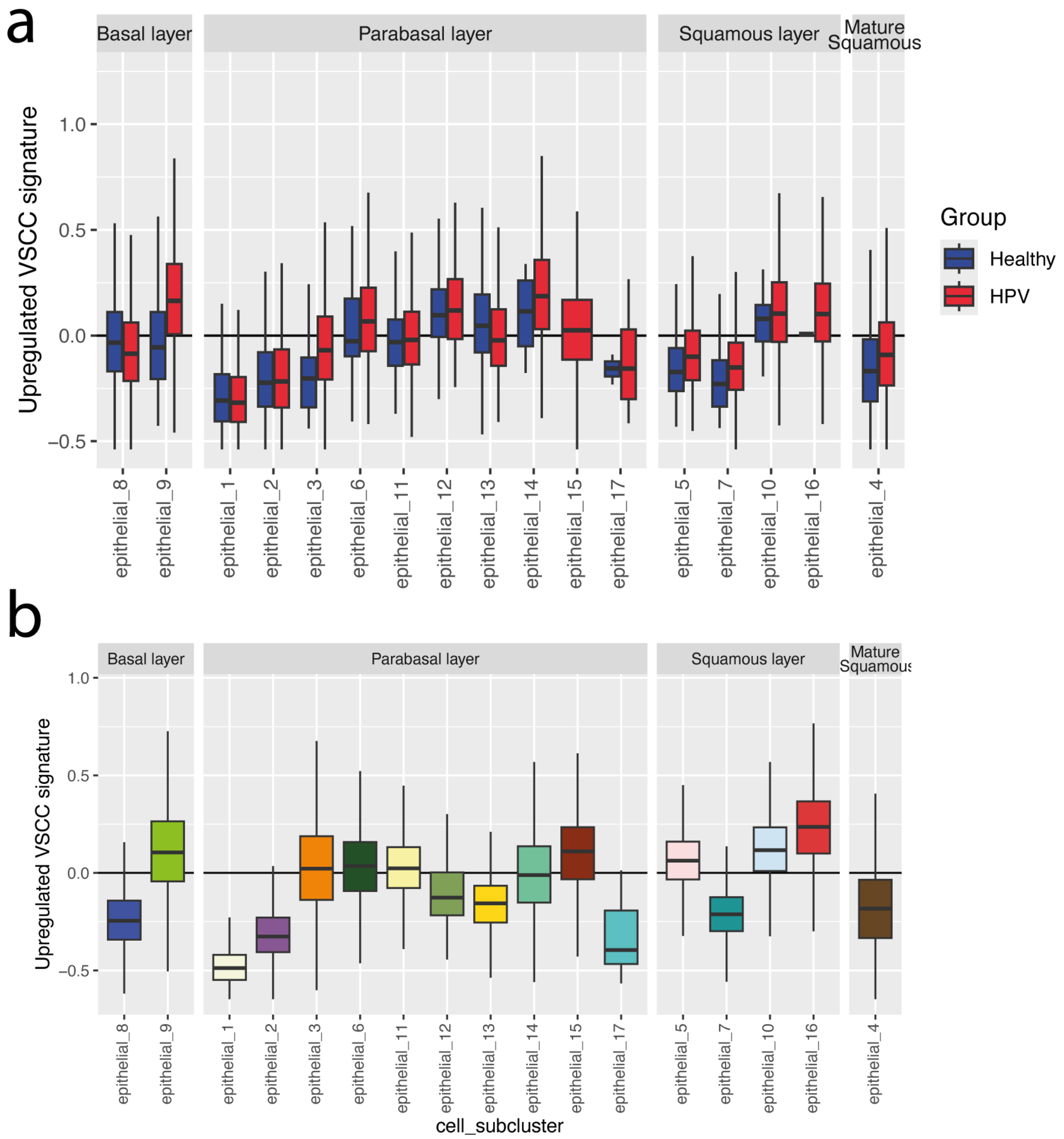

Figure S6 Comparison of VSCC transcriptomic signatures to individual epithelial subcluster signatures. a Transcriptomic signature of HPV-positive and -negative VSCC based on differentially expressed genes when compared to healthy vulva after reanalysis of the data from Micci et al.[10] applied to epithelial subcluster signatures from HPV16 vHSIL. Blue healthy epithelium, red epithelial cells from HPV epithelium. **b** Signature of the HPV+ VSCC based on differentially expressed genes when compared to HPV- VSCC applied to epithelial subcluster signatures from HPV16+ vHSIL.

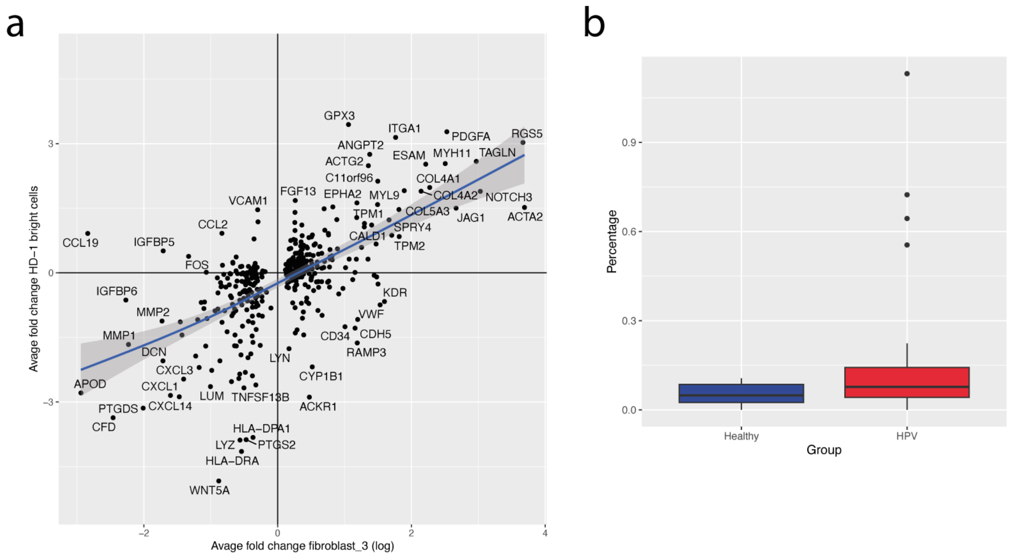

Figure S7. a Comparison of signature genes of fibroblast 3 versus that of pericytes from the study of Paquet-Fifield[12]. **b** Comparison of MDSCs in healthy and HPV-epithelium.

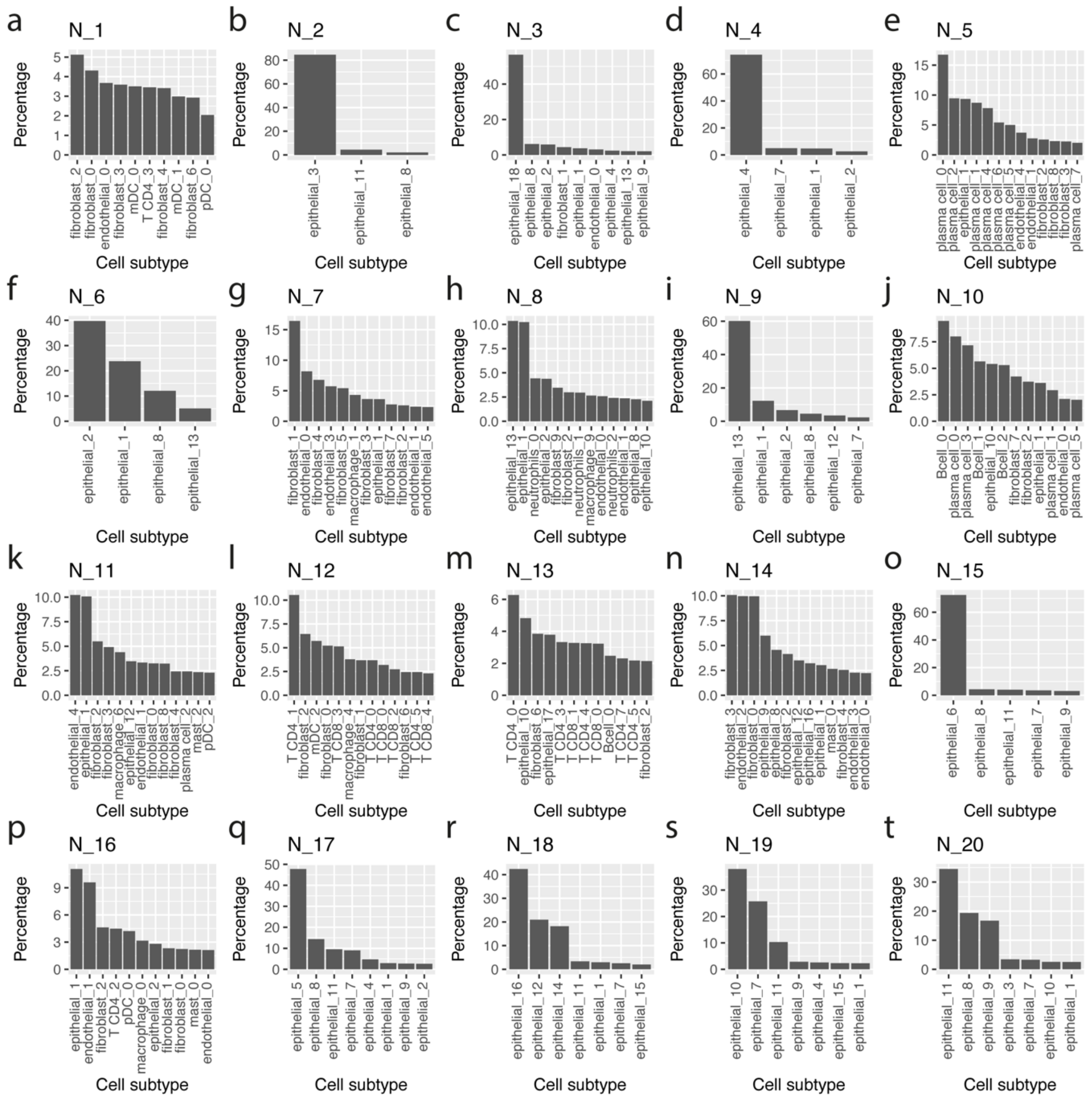

Figure S8 Specific cellular neighborhoods within healthy and vHSIL vulvar tissues. Neighborhoods of cells were identified across individuals. For each cell the 30 nearest neighboring cells were combined in a scaled sum of cell labels neighboring a given cell type. On these data, k-means clustering was performed resulting in 20 neighborhoods. Only cell types that represented at least 2% of the niche are shown in plots.

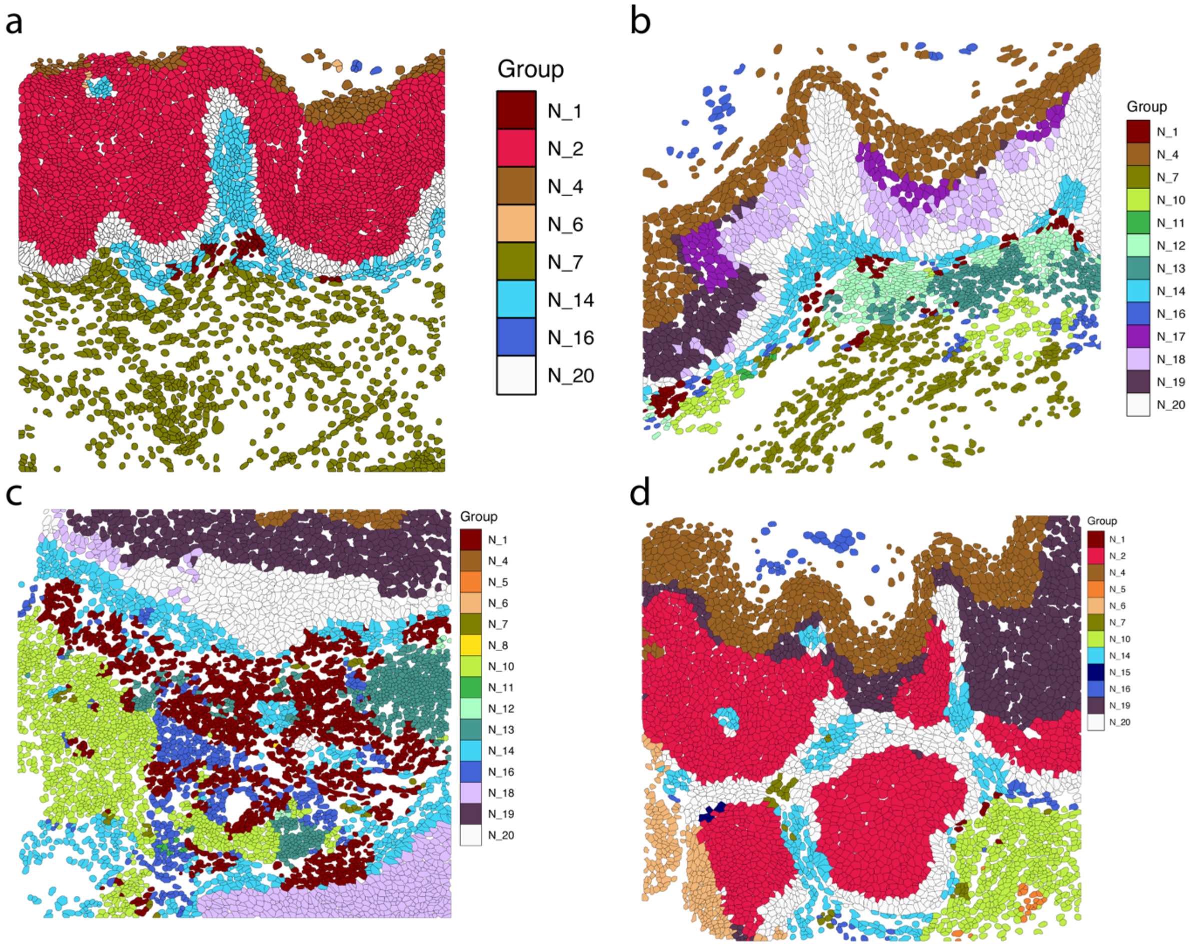

Figure S9. Examples of cellular neighborhoods detected in multiple patients. Cell present in each of the neighborhoods are indicated in Figure S7.

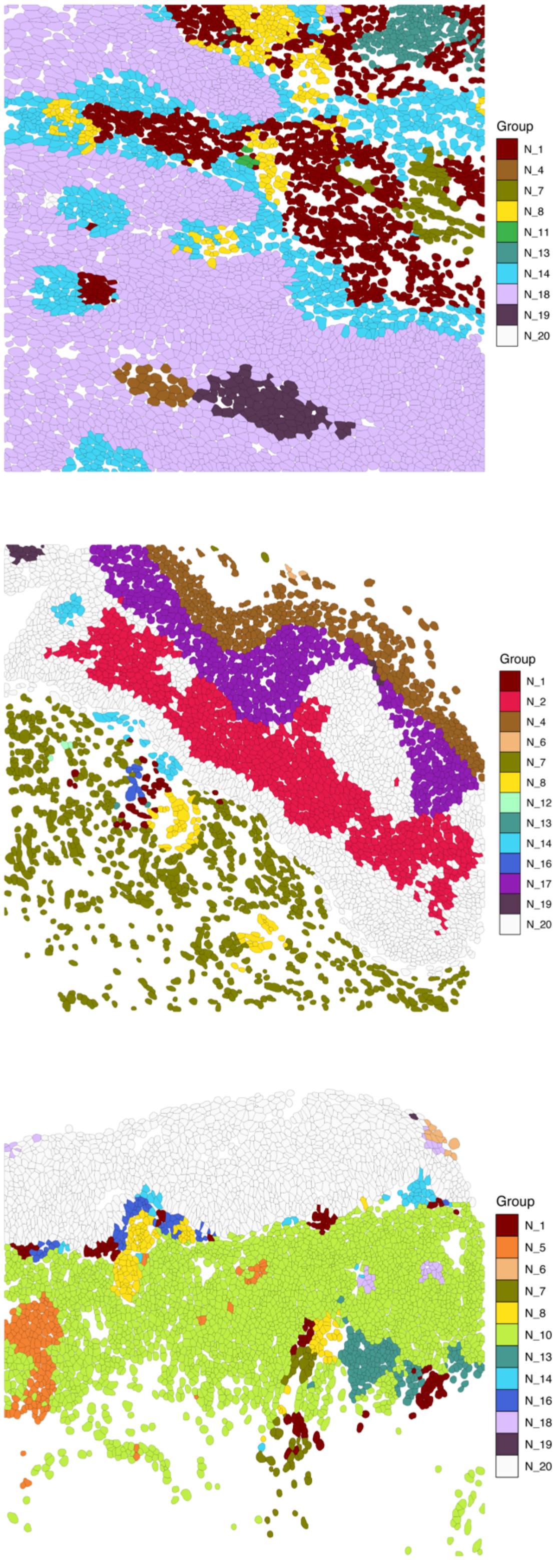

Figure S10. Examples of the neutrophil rich neighborhood 8 in three patients.
